# The relationship between local field potentials (LFPs) and the electromagnetic fields that give rise to them

**DOI:** 10.3389/fnsys.2014.00233

**Published:** 2014-12-12

**Authors:** Colin G. Hales, Susan Pockett

**Affiliations:** ^1^Neuroengineering Laboratory, Department of Electrical and Electronic Engineering, University of MelbourneCarlton, VIC, Australia; ^2^School of Psychology, University of AucklandAuckland, New Zealand

**Keywords:** brain electromagnetism, local field potential, Maxwell's equations, scalar potential, vector potential

Recently there has been a call (Reimann et al., 2013) for a re-evaluation of the genesis of local field potentials (LFPs), a measurement deeply correlated with normal and pathological excitable cell tissue operation (Einevoll et al., 2013; Friston et al., 2014). The lack of a full scientific account of LFP origins additionally means that brain augmentation hardware, a primary tool for which is the manipulation of LFPs, is in effect pulling unmarked levers. How can we knowledgeably control LFPs when LFP origin itself is a mystery? Here we investigate how the task of revisiting LFP origins might best be approached.

LFPs originate in the two deeply interconnected fundamental physical fields of the brain: the vector electric field [**E**(**r**,t), V/m] and the vector magnetic field [**B**(**r**,t), V-s/m^2^]. Each of these can be Helmholtz-decomposed into the gradient of a scalar potential [say Φ(**r**,t)] and the curl of a vector potential [say **A**(**r**,t)] (Groot and Suttorp, 1972; Landau et al., 1984; Malmivuo and Plonsey, 1995; Jackson, 1999). This means in practice that there are three “potential fields” operating in the brain^1^. At present it is technologically impossible to directly measure the vector electric field or magnetic field at the resolution of tissue fine structure. Therefore neuroscientists rely on a technically straightforward measurement of voltage (call it LFP(**r**,t)) that imperfectly accesses the “potential fields” and within which **E** and **B** are only indirectly represented.

Empirical work over many decades has converged on transmembrane ionic current as the ultimate origin of the LFP (Buzsaki et al., 2012; Destexhe and Bedard, 2013). This means we must address the finest details of the formidably complex tissue ultra-structure typified by Figure 1A (Nicholson and Sykova, 1998; Briggman and Denk, 2006; Kinney et al., 2013)^2^. This is because the ionic currents originate in the membrane micro-environment indicated by the generic sources d1·sd4 in Figure 1A. Fundamental field theory tells us that **E** and **B** actually mediate LFP expression. This requires us to look at how membrane-related sources first cause **E** and **B** and through them, the LFP. We must treat transmembrane currents and their supporting systems of charge as electromagnetic (EM) field sources.

**Figure 1:**
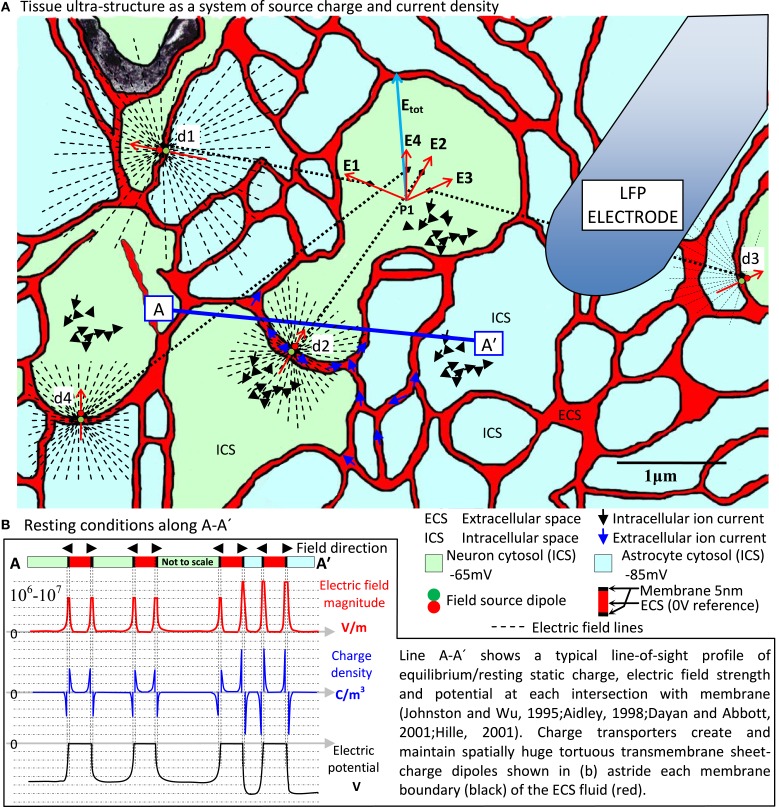
**EM field origins in nervous tissue ultra-structure**. **(A)** Electron micrograph colored to reveal neuron/glia ultra-structure with **(B)** the resting state source charge density characteristic centered on the huge transmembrane electric field (10^6^–10^7^ V/m) across all neural and glial cell membrane and maintained by charge transporters not shown. This massive sheet-charge dipole lines the tortuous, narrow sheet/tunnel ECS (Kinney et al., 2013), which is the only tissue medium actually outside all cells. Spatially and temporally coherent ion channel activity in neuronal membranes produces fast, coherent, dynamic current sources that locally modulate (even reverse) the planar dipole field, expressing dynamic electric and magnetic field systems far into the surrounding tissue. This is the primary source that originates all other activity in the tissue. At any given point (say P1) there is a total electric and magnetic field expressed line-of-sight through the tissue at the speed of light. This total field exerts its influence on local charge populations via the Lorentz force. Secondary current systems in the ECS (blue arrows) and ICS (black arrows) resulting from this activity are hugely diluted, diffuse and randomized, traveling at speeds 10,000 times slower than through the membrane (Hille, 2001). Such a small, randomized current density cannot be argued to contribute anything more than field noise at the scale of tissue ultra-structure. However, long term persistent charge transport can support regional polarization and thereby cause the tissue as a whole to exhibit a macroscopic electric field system. In this way, an ultra-structured EM field system and a large-scale slow electric field system can operate simultaneously in the tissue. It is also a natural expectation of such a system that all EM field sources (probably minutely) influence, through the tissue at the speed of light, all other field sources. This is the probable origin of the recently revealed EM field coupling mechanism (Frohlich and Mccormick, 2010; Anastassiou et al., 2011). **(A)** Based on (Nicholson and Sykova, 1998; Kinney et al., 2013), neuron/astrocyte allocation notional.

LFP(**r**,t) measurement arose as a lab technique nearly 70 years ago (Brooks and Eccles, 1947) and still involves insertion of electrodes that are huge compared to the cyto-architectural scale of the tissue. These electrodes inevitably disrupt the structure around their insertion routes and the eventual measurement points, homogenizing the tissue to some extent and causing an inflammatory response that adds to the disruption. Thus a localized artificial medium is created around each electrode tip, which forms the actual context of the LFP(**r**,t) measurement. The measurement reveals a spatial average (dependent on the electrode tip geometry) and a temporal average (dependent on sample rate and filters in the measurement equipment) voltage differential relative to a reference electrode elsewhere in the tissue. LFP(**r**,t) cannot be automatically claimed to access the scalar electric potential Φ(**r**,t) in the natural tissue. Even if contributions from tissue damage can be ignored, we are not directly measuring Φ. Rather, we are measuring some spatiotemporal average of Φ, the nature of which is not obvious and gets little attention in the literature. This LFP ⇔ Φ mapping needs to be revisited as part of a campaign of elucidating LFP origins.

Another important factor affecting the ability to infer EM fields from voltage measurements is that there are an infinity of different **E** and **B** fields that can give rise to the same Φ (and therefore the same LFP). This degeneracy of Φ owes its mathematical origin to what is called, in classical electromagnetism, electromagnetic gauge (Jackson, 1999). **E** and **B** are not uniquely revealed by Φ. Scalar electric potential Φ is like a height measurement. The lack of specificity that scalar potential has as a reflection of the electric field generating it is analogous to the degeneracy that height has to the terrain. If I have a height of 20 m, am I on my balcony or up a tree? Thus LFPs cannot be properly interpreted or understood without a good theoretical foundation for the origins of **E** and **B** based on real tissue ultra-structure knowledge. The LFP is a one-way lens. **E** and **B** can “see” Φ but Φ cannot “see” **E** and **B**. A practical example of the degeneracy of Φ is in the use of lumped-element circuit models of neurons. These models accurately replicate voltages and currents even though the field (**E** and **B**) system of the model is totally unlike that of real tissue. This technique confers a degree of useful predictive utility, but loses contact with the actual underlying tissue physics. The degeneracy in potentials is the reason we can abstract-away **E** and **B** physics and is central to the success of circuit theory (Plonsey and Collin, 1961, p. 326). However, degeneracy in electric potentials means that the EM field system implicit in a tissue's circuit-element model cannot be claimed to be the EM field system of the tissue.

## From charges to EM fields to LFPs

**E** and **B** sources are simply expressed by Maxwell's equations. An aggregate primary source “charge density (scalar) field” ρ(**r**,t) (C/m^3^) impresses an electric field system on space well beyond its bounds (notionally to infinity) by line of sight and at the speed of light. If a subset of that *same set of charges* happens to move and thereby create a primary “current density (vector) field,” **J**(**r**,t) (A/m^2^), then this charge motion (1) disturbs the charge density field, modulating the electric field commensurate with the spatial and temporal scale and detail of the changes, and (2) creates a magnetic field by virtue of the current density field. This is a universal property of Maxwell's equations.

In tissue, **E** and **B** owe their origins to the massive transmembrane sheet-charge density dipole astride all cell boundaries (Figure 1B), which dominates all other atomic/molecular sources. Neuron transmembrane disturbances in the sheet dipole charge density then dominate EM field dynamics. So at least at this level, **E** and **B** origins are easy to find. Detailing them, however, is the big challenge.

When attempting to meet this challenge, it is important to remember that in tissue, **E** and **B** are causally *prior*. Every kind of current and voltage elsewhere in the tissue is secondary. For example, consider the primary sources d1·s d4 shown in Figure 1A. Vector superposition creates electric field **E_tot_** at point P1 and via the Lorentz force this produces a *secondary* current in the tissue at P1, which has nothing directly to do with the current at the sources d1·s d4. Independent vector superposition of **E** and **B** means each field is a unified, emergent single entity with a spatiotemporal life and a causal influence of its own.

## Sources: density, coherence and persistence are masters

In Maxwell's equations **E** and **B** are intrinsically connected to current *density*, not current. Consider a single current that is first in the form of (i) fast, highly aligned transmembrane current filaments that then become (ii) slow, randomized and diffuse in the intracellular space (Figure 1A, black arrows). At some distant point the current operating in form (i) will impress a dominant, coherent EM field system whereas form (ii) will only create relative field “noise.” The spatial (tightly co-located ion channels) and temporal (all firing at once) coherence of the transmembrane part of the ion transport means that field contribution (i) will dominate. This is how charge and current densities collocated and aligned in space, and aligned in time will result in dominant **E_tot_** and **B_tot_** vectors with functional consequences (consistent pointing, rotating, pulsing). Non-coherent source contributions result in **E_tot_** and **B_tot_** noise.

Additionally, persistent synchronous vector electric field expression by cells and cell assemblies can slowly move large populations of charge to create regional charge densities. The resultant electric field “atmosphere” superposes (feeds back) vectorially onto all endogenous EM field ultra-structure sources. Yet none of it would exist were it not for the source systems and dynamics expressed at the level of the Figure 1A tissue ultra-structure. The electric and magnetic field system therefore has an extraordinarily deep spatial and temporal structure, all of which involves itself in what is seen as the LFP. We are thereby forced to accept that cell and cell assembly signaling is deeply involved at the ultra-structure level of EM field expression. This means the EM fields have 6–8 orders of magnitude of spatiotemporal detail (neural membrane to whole tissue) and that fully understanding LFP means characterizing tissue with models incorporating that level of depth.

## Configuring Maxwell's equations

The configuration of Maxwell's equations applicable at the level of the Figure 1A neuron transmembrane microenvironment, where **E** and **B** originate, also needs to be revised. This is necessary because the applicable charge transport equations are, technically, convection (Kirby, 2010). Convection current occurs when charge flows through an insulating medium such as liquid, rarefied gas or a vacuum (Sadiku, 2001, p. 163). ECS/ICS electrolyte currents are ions (charge) flowing in water, which is an extremely good insulator. Transmembrane ions travel through protein pores that have the same status as water at the time. Therefore convection is the applicable form of charge transport in ECS, ICS and through the membrane. How convection differs from formal conduction can be understood in terms of how charge density involves itself in charge transport dynamics. Convection involves using charge-density-dependent ion mobility properties and diffusion rather than charge-density-independent conductivity (Hille, 2001). Formal conduction involves charge motion under conditions of zero charge density maintained at the atomic scale. This happens in crystalline solid electron/hole conduction (Jackson, 1999, p. 706). In contrast, convective atomic ion transport can express a net charge density as it flows.

Yet conduction formalisms such as Ohm's Law are effective at quantifying currents and voltages in an overall sense of action potential signaling and LFP usage in the lab. This is because at spatial scales above the neuron membrane microenvironment, the regional average charge density asymptotes to zero. In the brain this is called “electro-neutrality” (Johnston and Wu, 1995; Nunez and Srinivasan, 2006). There is an overall balance in ion charge species in the brain. But that overall balance includes a radically dynamic imbalance around the membrane—otherwise there would be no resting potential, no neuronal signaling and no EM field expression.

Therefore any form of reconfigured Maxwell's equations must include a formal reconciliation between (1) the non-Ohmic nano-scale convection/diffusion charge transport proximal to/inside the membrane that originates the **E** and **B** fields, with (2) the charge-neutral conditions obviously amenable to conduction formalisms that exist at scales above the membrane/ECS microenvironment, which have no bearing on **E** and **B** origination, but are consistent with it in a voltage/current sense. A future accurate formalism is one that originates both **E** and **B** using convection/diffusion processes, which then asymptotes seamlessly to the more familiar conduction formalisms at some spatio-temporal scale to be determined. The new view and the old can thereby meet in a familiar way.

With microscopic **E** and **B** formalized and the important LFP ⇔ Φ mapping (electrode/tissue interaction) understood, years of LFP measurements become a revitalized body of evidence. Historically challenging concepts such as “open/closed field” (Nadasdy et al., 1998; Buzsaki et al., 2012), “neural field” (Coombes, 2006; Pinotsis and Friston, 2014), “power law spectra” (Buzsáki and Draguhn, 2004) and “ephaptic coupling” (Frohlich and Mccormick, 2010; Anastassiou et al., 2011) may take their mature form.

## Conclusion

The critical path to successful hardware-based brain augmentation requires us to heed a recent call to revisit the genesis of the LFP. In the present paper, a broad-brush review reveals ways for physicists and neuroscientists to meet productively to that end. The primary need is to attend to the genesis of the electric and magnetic fields of the brain at the level of tissue ultra-structure, via spatiotemporally coherent systems of source charge density and source current density centered on the neural membrane. The configuration of Maxwell's equations also needs rework. The degeneracy in potentials inherent in Maxwell's equations has been a historical misdirection in EM field understanding. The ultra-structural basis of the EM fields, embedded in cell and cell assembly activity, is a productive route to understanding EM field effects at all the usual spatiotemporal scales examined in the lab. Only then can these fields reveal the true nature of the measurement we call the LFP.

### Conflict of interest statement

The authors declare that the research was conducted in the absence of any commercial or financial relationships that could be construed as a potential conflict of interest.

## Footnotes

^1^The lack of a “magnetic monopole/charge” eliminates the scalar magnetic potential component of **B**(**r**,t), leaving **B** entirely characterized by a “vector potential,” which can be mathematically related to the electric vector potential decomposition of the electric field **E** (Jackson, 1999).

^2^Tissue ultra-structure refers to tissue details as revealed when imaged at Angstrom (10^−10^ m) resolution, usually with electron microscopy.
